# Potential Anatomical Implications of Filum Terminale Paraganglioma on Coccygodynia: A Case Report

**DOI:** 10.7759/cureus.25308

**Published:** 2022-05-24

**Authors:** Dimosthenis Rammos, Dimosthenis Chrysikos, Panagiotis Giavopoulos, Iordanis Alexiadis, Spyridon Theodoropoulos

**Affiliations:** 1 General Surgery, General Hospital of Piraeus "Tzaneio", Piraeus, GRC; 2 Department of Anatomy, Medical School, National and Kapodistrian University of Athens, Athens, GRC; 3 Neurological Surgery, General Hospital of Piraeus "Tzaneio", Piraeus, GRC

**Keywords:** intradural tumor, spinal tumor, paraganglioma, filum terminale, coccygodynia

## Abstract

In this case report, we present a unique cause of coccygodynia due to an intradural lumbar spinal tumour in a patient with multiple filum terminale paragangliomas. We highlight the symptomatology, the clinical course, and the radiological findings. Our review of the literature proved our case to be the first report of an intradural cauda equina tumour presenting with coccygodynia in English literature. Based on the outcome and clinical response to treatment we make a bold hypothesis on the possible anatomical mechanism of his coccygodynia.

## Introduction

Paragangliomas are neoplasms originating from the autonomic nervous system, found generally in adrenal and extra-adrenal locations. Extra-adrenal paragangliomas are rare and occur most commonly in the carotid bodies and the jugular glomus. Primary spinal paragangliomas are extremely rare, most frequently involving the cauda equina and the filum terminale [1]. The most common symptom of filum terminale paragangliomas is back pain with or without radiculopathy as well as neurological symptoms such as paraparesis [2]. Our case represents a unique manifestation for an intradural cauda equina tumour since our patient presented with coccygodynia with no sciatic pain or neurological symptoms. In this case report, we highlight on his symptomatology, radiological findings, clinical course and response to surgical treatment and based on the outcome suggest a hypothesis on the mechanism of his coccygodynia.

## Case presentation

A 47-year-old male patient was referred to our Neurosurgical Department due to a two-year-long history of coccygodynia not responding to pain management. The patient was assessed multiple times by General Practitioners and Orthopaedic surgeons during all that period and was finally referred for a pelvis MRI. Due to an abnormal finding, he underwent a complementary lumbar spine MRI and then was referred to our department.

The patient was complaining of pain in his tail bone area, sometimes aggravated in the morning and he described no particular activities or positions to alleviate or provoke the pain. Especially, he did not seem to be affected by prolonged sitting or pressure in the area. Clinical and neurological examinations were unremarkable whereas palpation and manipulation of the coccyx did not reveal any tenderness. The patient had no past medical history and did not report any recent injury.

**Figure 1:**
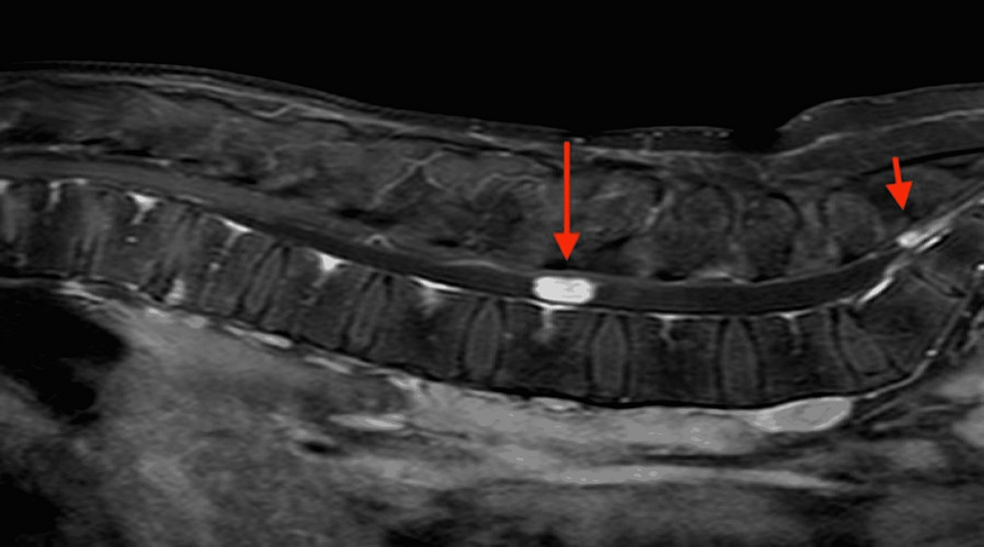
Sagittal T1 Gd+ MRI of the lumbar spine Sagittal T1 Gd+ MRI of the lumbar spine showing two intradural cauda equina tumours, a larger one at the L3 level (big arrow) and a smaller one at the S2 level (small arrow).

**Figure 2:**
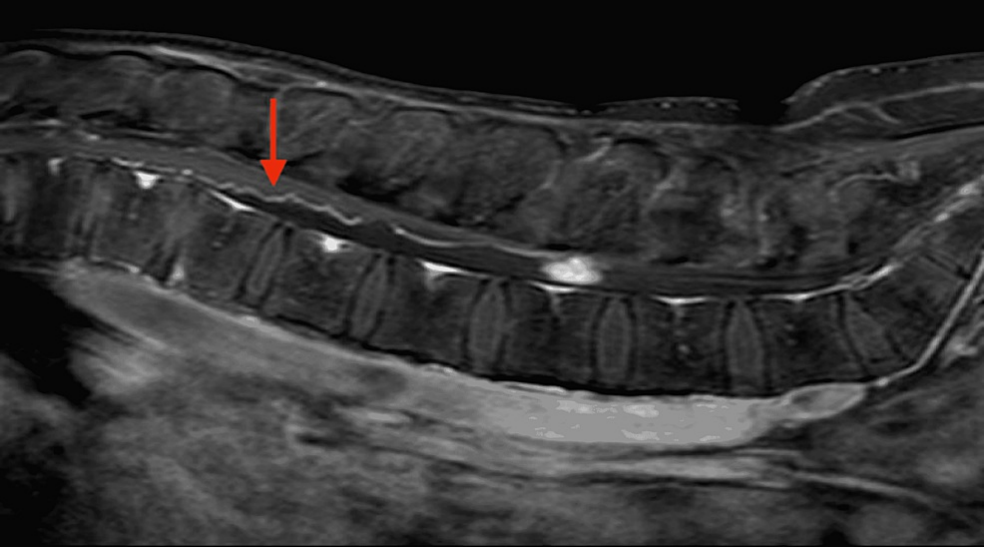
Sagittal T1 Gd+ MRI of the same patient Sagittal T1 Gd+ MRI of the same patient disclosed abnormal, serpiginous vessels between the tumour at L3 and the conus medullaris (arrow). This represents a typical sign of filum terminale paragangliomas.

**Figure 3:**
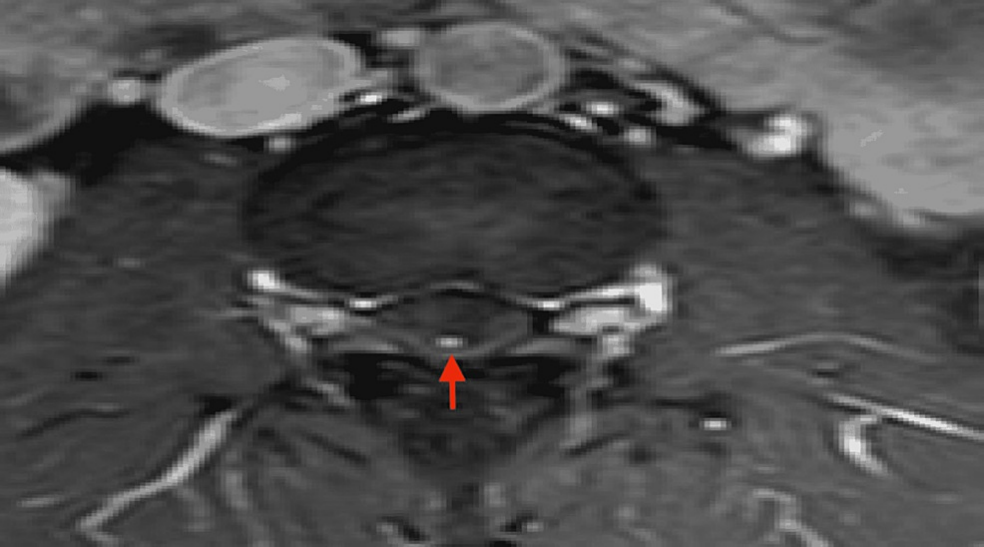
Axial T1 Gd+ MRI Axial T1 Gd+ MRI above the superior end of the tumour shows a large intrathecal vessel that continues rostrally (arrow), indicative of a hypervascular lesion.

MRI of his lumbar spine revealed a large intradural, homogeneously enhancing tumour at the level of the L3 vertebra, as well as a smaller one with similar radiological characteristics at the level of S2 (Figure 1). A characteristic finding in the MRI was the presence of serpentine, abnormal vessels between the conus and the large lesion at L3 indicative of a hypervascular lesion (Figures 2, 3). Prior to any intervention, the patient underwent a full MRI of his neuraxis that showed no other lesions.

After careful evaluation of the MRI findings, we made a decision to operate on the large one and based on the histology and clinical response to decide on further treatment. The patient underwent an L3 laminectomy and successful total excision of the intradural lesion through a standard durotomy. Intraoperatively, the tumour was well encapsulated, vascular and firmly attached to the filum terminale. A large vein was noted running adhered to its surface, few nerve roots were loosely adhered but easily dissected off the tumour and prominent bleeding was noted if the capsule was violated. The tumour was carefully dissected off surrounding nerve roots, the filum terminale was coagulated and then cut proximally and then distally achieving complete removal of the tumour. Postoperatively, the patient had an excellent recovery and complete resolution of his coccygodynia was reported.

Histological examination of the specimen revealed an encapsulated tumour with an alveolar pattern consisting of chief cells with granular, eosinophilic cytoplasm and spheroidal nuclei arranged in clusters separated by fine fibrovascular septae with dilated vessels (Zellbaten pattern). No nuclear atypia or necrosis was noted. Immunochemistry labeling was chromogranin (+), synaptophysin (+), S100 (+), GFAP (-), and keratin CK18.8 (+) confirming the diagnosis of a filum terminale paraganglioma.

**Figure 4:**
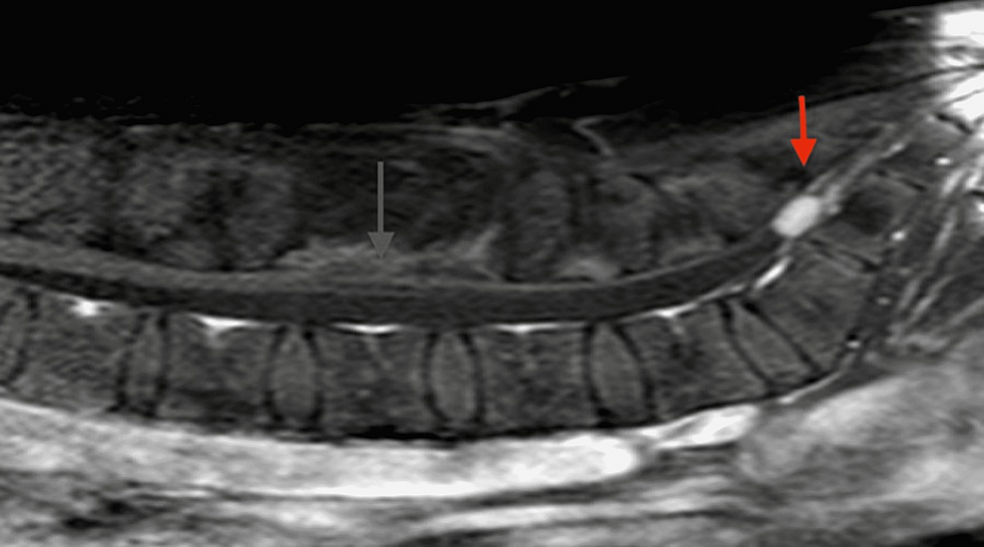
Sagittal post-operative T1 Gd+ MRI Sagittal post-operative T1 Gd+ MRI showing complete removal of the tumour at L3 (grey arrow) and increase in the size of the smaller one at S2 (red arrow), at the time of patient’s coccygodynia relapse.

Interestingly, the patient was under close follow-up and reported relapse of his coccygodynia approximately 18 months postoperatively. A new MRI was performed which revealed total removal of the tumour at L3 with no local recurrence but increase in size of the smaller lesion at S2 (Figure 4). The patient is scheduled for elective surgery to remove the second lesion.

## Discussion

Filum terminale paragangliomas are rare benign tumours that arise from the ectopic sympathetic neurons and become misplaced on the cauda equina rootlets or on the filum terminale [3,4]. Paragangliomas are slow-growing, well demarcated, intradural, and hypervascular tumours that neither infiltrate the conus nor the rootlets [5]. Rahimizadeh et al. [2] performed a recent literature review from 2009 until October 2020 and found 104 new cases reported, adding these cases to a previous review by Gutenberg et al. [5] to a total of 319 cases of filum terminale paragangliomas. The most common presenting symptom is low back pain with or without radiculopathy as with most intradural extramedullary tumours [2].

On the other hand, coccygodynia is a well-known clinical entity with variable aetiology most commonly attributed to major or repetitive minor trauma of the coccyx. Non-traumatic causes of coccygodynia include hypermobility or hypomobility of the sacrococcygeal joint, infections, neoplasms, degenerative joint disease, anatomical variants of coccygeal morphology, or even somatization disorder and other psychological disorders. The hallmark of coccyx pain in local causes is the typical pain that worsens with prolonged sitting and even with defecation and sexual intercourse. Physical examination usually reveals tenderness over the coccyx and pain on manipulation [6,7].

However, atypical coccygodynia could represent a referred or radicular pain and should prompt further evaluation of the patient [6]. In terms of lumbar spine pathology, lumbar disc herniations could cause coccygodynia [8] and there are even reports of arachnoid cysts and Tarlov's cysts as extremely rare causes of pain [9,10].

To our knowledge so far, there was no correlation between an intradural lumbar spine tumour presenting with coccygodynia. Therefore, we conducted a PubMed search using the terms coccygodynia/coccydynia/coccalgia, tailbone pain/coccyx pain/coccygeal pain, AND intradural tumour/spinal tumour or using specific tumour terms. According to our search, there were only two French articles in 1967 and 1968 reporting an ependymoma and a giant tumour of the cauda equina presenting with coccygodynia [11,12]. Our case represents the only case report of an intradural tumour presenting as coccygodynia in English literature so far.

In this context, we attempted to shed light on the possible correlation of such a tumour causing coccyx area pain. Based on the complete resolution of pain after removal of the large L3 tumour, we hypothesized that most probably this was a referred pain alleviated by removal of the lesion and transection of the filum terminale. We assumed that the tumour was causing mechanical strain on the filum terminale, which terminates attached to the first segment of the coccyx, potentially causing referred pain.

However, in our case, the patient experienced a relapse of his pain and underwent an MRI that revealed an increase in the size of the second smaller intradural S2 tumour and no recurrence at the original site of surgery. Obviously, this means that our original assumption was wrong and most probably the aetiology of pain should be radicular in nature and caused by compression of the lower sacral nerve roots whose dermatomes correspond to the area patients may describe as the tail bone area.

**Figure 5:**
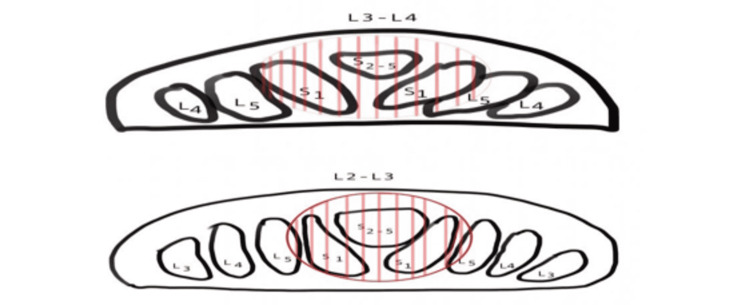
Schematic drawing of the intrathecal topographic organization of the cauda equina nerve roots at L2/L3 and L3/L4 levels. Hatch circle represents the approximate location of the filum terminale tumor. The drawing is based on the monumental human cadaveric study by Wall et al. (1990) [13]

Wall et al. described the cross-sectional anatomy of the cauda equina in 15 fresh human cadavers and discovered a highly consistent pattern [13]. The lower sacral (S2-S5) and coccygeal nerve roots were located in the dorsal aspect of the thecal sac just posterior to the filum terminale (Figure 5). Therefore, these rootlets are in close proximity to a lesion of the filum terminale and are more susceptible to injury due to direct compression by the tumour than the lumbar nerve roots. Moreover, the fact that the lower sacral and coccygeal nerve roots are thinner, could possibly make them more sensitive to compression injury. On the other hand, cauda equina nerve roots derive some of their nutritional supply via diffusion from the surrounding cerebrospinal fluid and any adherence to the tumour capsule could potentially hamper the diffusion mechanism and render them more susceptible to injury [14].

## Conclusions

Coccygodynia is a well-known entity with characteristic pain patterns and clinical signs. However, as highlighted by our case, first-line physicians should be aware of atypical coccygeal pain and suspect a referred or radicular pain mechanism so that they further evaluate patients and conduct a more thorough paraclinical investigation. Our case highlights such a paradigm and also demonstrates a unique clinical presentation of an intradural lumbar tumour.

## References

[1] Kalani MA, Chang SD, Vu B (2015). Paraganglioma of the filum terminale: case report, pathology and review of the literature. Cureus.

[2] Rahimizadeh A, Ahmadi SA, Koshki AM, Rahimizadeh A, Karimi M (2021). Paraganglioma of the filum terminal: case report and review of the literature. Int J Surg Case Rep.

[3] Aghakhani N, George B, Parker F (1999). Paraganglioma of the cauda equina region--report of two cases and review of the literature. Acta Neurochir (Wien).

[4] Miliaras GC, Kyritsis AP, Polyzoidis KS (2003). Cauda equina paraganglioma: a review. J Neurooncol.

[5] Gutenberg A, Wegner C, Pilgram-Pastor SM, Gunawan B, Rohde V, Giese A (2010). Paraganglioma of the filum terminale: review and report of the first case analyzed by CGH. Clin Neuropathol.

[6] Nathan ST, Fisher BE, Roberts CS (2010). Coccydynia: a review of pathoanatomy, aetiology, treatment and outcome. J Bone Joint Surg Br.

[7] Lirette LS, Chaiban G, Tolba R, Eissa H (2014). Coccydynia: an overview of the anatomy, etiology, and treatment of coccyx pain. Ochsner J.

[8] Patijn J, Janssen M, Hayek S, Mekhail N, Van Zundert J, van Kleef M (2010). 14. Coccygodynia. Pain Pract.

[9] Kepski A, Rudnicki S (1978). Arachnoid cyst of the cauda equina: a contribution to the etiology of coccygodynia (Article in Polish). Neurol Neurochir Pol.

[10] Dehaine V, Wechsler B, Ziza JM (1990). Coccygodynia disclosing Tarlov's cysts (Article in French). Rev Med Interne.

[11] Charpentier J, Messimy R, da Lage C (1968). Coccygodynia revealing an ependymoma of the filum terminale. Complete removal without sequelae (Article in French). Rev Neurol (Paris).

[12] Chateau R, de Rougemont J, Bonneville B, Barge M, Groslambert R, Perret J (1967). Coccygodynia revealing a giant tumor of the cauda equina (Article in French). J Med Lyon.

[13] Wall EJ, Cohen MS, Massie JB, Rydevik B, Garfin SR (1990). Cauda equina anatomy. I: intrathecal nerve root organization. Spine (Phila Pa 1976).

[14] Rydevik B (1993). Neurophysiology of cauda equina compression. Acta Orthop Scand Suppl.

